# The Contribution of *SAA1* Polymorphisms to Familial Mediterranean Fever Susceptibility in the Japanese Population

**DOI:** 10.1371/journal.pone.0055227

**Published:** 2013-02-20

**Authors:** Kiyoshi Migita, Kazunaga Agematsu, Junya Masumoto, Hiroaki Ida, Seiyo Honda, Yuka Jiuchi, Yasumori Izumi, Yumi Maeda, Ritei Uehara, Yoshikazu Nakamura, Tomohiro Koga, Atsushi Kawakami, Munetoshi Nakashima, Yuichiro Fujieda, Fumiaki Nonaka, Katsumi Eguchi, Hiroshi Furukawa, Tadashi Nakamura, Minoru Nakamura, Michio Yasunami

**Affiliations:** 1 Clinical Research Center, Department of General Internal Medicine and NHO Nagasaki Medical Center, Kubara, Omura, Japan; 2 Department of Infection and Host Defense Graduate School of Medicine, Shinshu University, Asahi, Matsumoto, Japan; 3 Department of Pathogenomics, Ehime University Graduate School of Medicine, Toone City, Ehime, Japan; 4 Department of Rheumatology, Kurume University School of Medicine, Kurume, Japan; 5 Department of Public Health, Jichi Medical University, Tochigi, Japan; 6 First Department of Internal Medicine, Nagasaki University School of Medicine, Sakamoto, Nagasaki, Japan; 7 Department of Rheumatology, Red Cross Nagasaki Atomic bomb Hospital, Mori, Nagasaki, Japan; 8 Department of Medicine II, Hokkaido University Graduate School of Medicine, Sapporo, Japan; 9 Department of Rheumatology, Sasebo City General Hospital, Hirase, Sasebo, Japan; 10 Department of Rheumatology, NHO Sagamihara Hospital, Sakuradai, Sagamihara, Japan; 11 Department of Rheumatology, NTT WEST JAPAN Kyushu Hospital, Shinyashiki, Kumamoto, Japan; 12 Center for International Collaborative Research (CICORN) and Institute of Tropical Medicine, Nagasaki University, Nagasaki, Japan; University of Thessaly, Greece

## Abstract

**Background/Aims:**

Familial Mediterranean Fever (FMF) has traditionally been considered to be an autosomal-recessive disease, however, it has been observed that substantial numbers of patients with FMF possess only 1 demonstrable *MEFV* mutation. The clinical profile of familial Mediterranean fever (FMF) may be influenced by *MEFV* allelic heterogeneity and other genetic and/or environmental factors.

**Methodology/Principal Findings:**

In view of the inflammatory nature of FMF, we investigated whether serum amyloid A (SAA) and interleukin-1 beta (IL-1β) gene polymorphisms may affect the susceptibility of Japanese patients with FMF. The genotypes of the -13C/T SNP in the 5′-flanking region of the *SAA1* gene and the two SNPs within exon 3 of *SAA1* (2995C/T and 3010C/T polymorphisms) were determined in 83 Japanese patients with FMF and 200 healthy controls. The same samples were genotyped for IL-1β-511 (C/T) and IL-1 receptor antagonist (IL-1Ra) variable number of tandem repeat (VNTR) polymorphisms. There were no significant differences between FMF patients and healthy subjects in the genotypic distribution of *IL-1β -511* (C/T), *IL-1Ra* VNTR and *SAA2* polymorphisms. The frequencies of *SAA1.1* allele were significantly lower (21.7% versus 34.0%), and inversely the frequencies of *SAA1.3* allele were higher (48.8% versus 37.5%) in FMF patients compared with healthy subjects. The frequency of -13T alleles, associated with the *SAA1.3* allele in the Japanese population, was significantly higher (56.0% versus 41.0%, *p* = 0.001) in FMF patients compared with healthy subjects.

**Conclusions/Significance:**

Our data indicate that *SAA1* gene polymorphisms, consisting of -13T/C SNP in the 5′-flanking region and SNPs within exon 3 (2995C/T and 3010C/T polymorphisms) of *SAA1* gene, are associated with susceptibility to FMF in the Japanese population.

## Introduction

FMF is an inherited autoinflammatory disease characterized by recurrent self-limited fever, and serositis [1]. These episodes of inflammation are mainly mediated by a massive influx of neutrophils into serous cavities and are accompanied by an elevation of acute phase reactants [2]. The disease is associated with mutations in the *MEFV* gene that encodes pyrin, and is transmitted in an autosomal-recessive manner [3]. Therefore, heterozygotes are expected to be carriers or lack the clinical phenotype of FMF. However, mutations in the second *MEFV* allele have not been observed in 20–30% of patients with typical FMF [4]. Recent studies suggest that subjects with a single *MEFV* mutation may cross a threshold for the development of an FMF phenotype if they also express a combination of gene polymorphisms that favor increased inflammation [5]. These polymorphisms are thought to belong to genes of the interleukin-1β/innate immune system pathways [5]. The IL-1 family of cytokines is critical to the host's response to infection, and induction of innate immunity and acute phase inflammation [6]. The overproduction of IL-1β is responsible for a variety of autoinflammatory syndromes including FMF [7]. IL-1β requires cleavage via caspase-1 for proper secretion, which is facilitated by inflammasome activation [8]. The NOD-like receptor family, pryin domain containing 3 (NLRP3) inflammasome has emerged as a critical cytosolic sensor for a number of endogenous mediators, including amyloid protein [9].

Recent studies have shown that serum amyloid A (SAA) induced the expression of pro-IL-1β and activated the NLRP3 inflammasome in a cathepsin B and P2X_7_-dependent manner resulting in secretion of mature IL-1β [10]. SAA is an acute-phase protein, which increases in the serum during inflammation and is susceptible to proteolytic cleavage to amyloid A (AA) protein, the major fibrillar protein in secondary amyloidosis [11]. An allelic variant of *SAA1.3*, was found to be associated with AA amyloidosis in Japanese rheumatoid arthritis (RA) patients [12]. In view of the recent genetic studies in FMF, other modifying genetic factors may contribute to the susceptibility or clinical expression of FMF in addition to *MEFV* mutations. Therefore, we attempted to determine the effect of gene polymorphisms on the susceptibility to FMF in the Japanese population.

## Materials and Methods

### Patients

In early 2007, a laboratory network collecting the genetic diagnosis of periodic fever was established at the Japan Autoinflammation Association (JAA), and *MEFV* gene analysis was carried out at the Clinical Research Center of National Hospital Organization (NHO) Nagasaki Medical Center. Up to October 2012, 481 consecutive unrelated patients with periodic fever were referred and underwent molecular diagnosis at the NHO Nagasaki Medical Center. All patient, who were originating from Japan, (East Japan n = 36, West Japan n = 47) were asked to complete a questionnaire that included demographics (sex, age of onset), family history (consanguinity of parents, family history of recurrent fever), and the presence of recurrent febrile attacks typical of FMF, including peritonitis, pleuritis, arthritis, and transient inflammatory responses. The genetic analysis of *MEFV* gene was approved by the Ethics Committee of Nagasaki Medical Center, and written informed consent was obtained from each individual. On the basis of Tel-Hashomer criteria [13], we divided the FMF patients in two groups: Group 1, typical FMF exhibiting the presence of 1 or more major criteria independent to the presence of minor criteria; Group 2, incomplete FMF exhibiting the absence of major criteria and 2 or more minor criteria. It is important to stress that response to colchicine was confirmed in almost all patients. As controls, 200 healthy Japanese individuals without pre-existing medical diseases (90 men and 110 women 14 to 64 years, with a mean age of 38.6±13.9 years) from East Japan (n = 86) and West Japan (n = 114) were enrolled in the study after obtaining informed consent.

### *MEFV* gene Mutation analysis

All patients were undergone genetic analysis of *MEFV* gene exons 1, 2, 3 and 10 by direct sequencing. 2 milliliters of blood samples were collected from all subjects. Genomic DNA was extracted from whole blood by means of the Promega Wizard® Genomic DNA Purification Kit (Promega, USA). Mutation analysis was performed by genomic sequencing as described previously [14].

### Genotyping

#### *SAA1* gene

The genotype of the SAA1 -13C/T in the 5-region of exon 1 (rs11024595) was determined by the polymerase chain reaction–restriction fragment length polymorphism (PCR–RFLP) method [15]. The primers used for the PCR reaction were 5′-ACATCT TGTTCCCTC AGGTTG-3′ (sense) and 5′-GCTGTAGCTGAGCTGCGG-3′ (antisense).

The 229-bp PCR products were digested with restriction enzyme AciI (BioLabs, Beverly, MA, USA) and electrophoresed on a 12.5% polyacrylamide gel [15].

The *SAA1.1, 1.3*, and *1.5* alleles, corresponding to the T-C, C-T, and C-C haplotypes of the C2995T (rs1136743) and C3010T (rs1136747) polymorphisms were also determined by the PCR-RFLP [15]. The primers used for the PCR reaction were 5′-GCC AATTACATCGGCCTCAG-3′ (sense) and 5′-TGGCCA AAGAATCTCTGG AT-3′ (antisense).

The 518-bp PCR products were digested with restriction enzyme BclI (Promega, San Luis Obispo, CA, USA) and BanI (Promega) and electrophoresed on a 2.5% agarose gel [15].

The genotype of the *SAA2* (rs2468844) was determined by the polymerase chain reaction–restriction fragment length polymorphism (PCR–RFLP) method. The primers used for the PCR reaction were 5′-AGAGAATATCCAGAGACTCACAGGC-3′ (sense) and 5′-CAGGCCAGCAGGTCGGAAGT-3′(antisense). The 115 bp PCR products were digested with the restriction enzyme Nco I. The digested products were separated by 3% agarose gels by ethidium bromide staining [15].

#### IL-1Ra

For the IL-1RA VNTR polymorphism, the region including variable numbers of identical 86-bp tandem repeats was amplified by PCR using the following primers: 5′-CTCAGCCAACACTCCTAT-3′ (sense) and 5′-TCCTGGTCTGCAGGTAA-3′ (antisense). PCR products of 240(allele 2, two repeats), 325 (allele 3, three repeats), 410 (allele 4, four repeats), and 500 bp (allele 5, five repeats) were distinguished by agarose gel electrophoresis [16].

#### IL-1B-511

A fragment containing the *AvaI* polymorphic site at promoter region -511 of the IL-1B gene was amplified by PCR. PCR was carried out with primers, forward primer 5′-GCCTGAACCCTGCATACCGT-3′ (sense). 5′-GCCAATAGCCCTTGTCT-3′ (antisense). Fragments were separated by electrophoresis on 3% agarose with ethidium bromide staining using appropriate commercially available size markers for comparison. The C allele was designated if two bands of 92 and 63 bp were obtained, and the T allele was designated if a signal band of the undigested 155 bp was obtained. Genotypes were designated as follows: C/C, two bands of 92 and 63 bp: C/T, three bands of 155, 92, and 63 bp; and T/T, a single band of 155 bp [16].

### Statistical Analysis

Results are expressed as mean±SD. Statistical analysis was performed with SPSS18 for windows (SPSS Statistics, Illinois). The statistical significance of differences between groups was calculated by either the chi-square test for categorical data and Mann-Whitney's U-test for quantitative data. Deviation from Hardy-Weinberg equilibrium was assessed using the SNPAlyze software ver. 7.0 (Dynacom, Yokohama, Japan). A *p* value of <0.05 was considered significant.

## Results

### Demographic data and MEFV genotypes

We diagnosed 83 subjects, all of Japanese origins, as FMF. Among these patients, 44 were diagnosed as typical FMF and 37 were diagnosed as incomplete FMF. The demographic data of the newly-diagnosed FMF patients are summarized in Table 1. The overall male: female ration in patients with FMF was 0.8 (37:46). In incomplete FMF patients, the more affected sex is female in contrast to typical FMF (Table 1). The mean age ± SD at diagnosis was 37.9±18.8 years. Age at diagnosis of patients with typical FMF was similar to those with incomplete FMF (36.2±18.2 and 39.9±19.6 years, respectively; *p* = 0.419; Table 1). By mutation analysis, the *MEFV* gene mutation could not be identified in 3 of 83 patients (3.6%). The distribution of the *MEFV* genotype was heterogenous. The most frequent genotype was M694I/E148Q, followed by E148Q/normal and E84K/normal. AA amyloidosis was histologically confirmed in 4 patients with FMF, whose genotypes were M694I/M694I *SAA1.5/15*, M694I/E148Q/L110P *SAA1.1/1.1*, M694I/E148Q/L110P *SAA1.3/1.5* and E148Q/R202Q/P369S/R408Q *SAA1.3/1.5*.

**Table 1:** *MEFV* genotypes, gender, and the presence of amyloidosis in 83 Japanese patients with FMF.

*MEFV* genotypes	n(%)	Typical	(Male/Female)	Incomplete	(Male/Female)	Amyloidosis	*p* value
M694I/M694I	4(4.8)	4	(1/3)			1	
M694I/normal	4(4.8)	4	(4/0)				
M694I/E148Q	13(15.7)	13	(10/3)				
M694I/P751L	1(1.2)	1	(0/1)				
M694I/E148Q/E148Q	1(1.2)	1	(0/1)				
M694I/E148Q/L110P	5(6.0)	5	(3/2)			2	
P369S/R408Q	4(4.8)			4	(1/3)		
E148Q/P369S/R408Q	3(3.6)			3	(0/3)		
E148Q/E148Q/P369S/R408Q	4(4.8)	2	(2/0)	2	(0/2)		
E148Q/R202Q/P369S/R408Q	1(1.2)			1	(1/0)	1	
E148Q/G304R/P369S/R408Q	1(1.2)			1	(0/1)		
E148Q/E148Q/P369S/P369S/R408Q/R408Q	1(1.2)			1	(0/1)		
E148Q/normal	12(14.5)	6	(3/3)	6	(1/5)		
R202Q/normal	2(2.4)	1	(1/0)	1	(0/1)		
G304R/normal	1(1.2)			1	(1/0)		
E148Q/E148Q	1(1.2)	1	(0/1)				
E148Q/L110P	6(7.2)	1	(0/1)	5	(1/4)		
E148Q/R202Q	1(1.2)	1	(0/1)				
E148Q/E148Q/L110P	3(3.6)	1	(1/0)	2	(2/0)		
E148Q/L110P/R202Q	2(2.4)			2	(0/2)		
E84K/normal	8(9.6)	5	(3/2)	3	(1/2)		
E84K/E148Q	1(1.2)			1	(0/1)		
E84K/G304R	1(1.2)			1	(0/1)		
Normal	3(3.6)			3	(1/2)		
Gender (Male/Fmale)			(28/18)		(9/28)		*P*<0.0001
Age (years)			36.2±18.2		39.9±19.6		*p* = 0.419
Total			46		37		

Data are expressed as number (percentage). ± ; standard deviation. *p* values were calculated with chis-square test for qualitative data and Mann-Whitney test for quantitative data.

### *IL-1β* and *IL-1Ra* gene polymorphism

The genotype frequencies of IL-1β-511 (C/T), and IL-1Ra VNTR polymorphisms in FMF patients and healthy subjects are summarized in Table 2. There were no significant difference in the frequencies of these polymorphisms between FMF patients and healthy subjects.

**Table 2:** Frequencies of the genotypes at the *IL*-*1β* -511, *IL-1Ra* and *SAA2* loci in patients with FMF and healthy subjects.

	FMF patients	Healthy subjects	*p* value
	n = 83(%)	n = 200(%)	
Genotype at *IL*-*1β* -511 locus			
C/C	27(32.5)	59(29.5)	χ^2^ = 0.934 *p* = 0.627
C/T	43(51.8)	100(50.0)	
T/T	13(15.7)	41(20.5)	
Genotype at *IL*-*1Ra* locus			
1/1	73(88.0)	167(83.5)	χ^2^ = 2.451 *p* = 0.857
1/2	5(6.0)	20(10.0)	
1/3	0	1(0.5)	
1/4	4(4.8)	7(3.5)	
2/2	0	2(1.0)	
2/4	1(1.2)	3(1.5)	
Genotype at *SAA2* locus			
A/A	62(74.7)	163(81.5)	χ^2^ = 2.338 *p* = 0.276
A/G	19(22.9)	35(17.5)	
G/G	2(2.4)	2(1.0)	

*IL-1β*; Interleukin-*1β .IL-1Ra*; Interleukin-1 receptor antagonist .*SAA2*; Serum amyloid A2. Chi-square test was used to examine differences of genotype and allele frequencies between FMF patients and healthy subjects.

### Association between *SAA2* gene polymorphism and FMF

There was no significant difference in the frequencies of the *SAA2* genotype between FMF patients and healthy subjects (Table 2).

### Association between *SAA1* gene polymorphisms and FMF

A segment of the genomic *SAA1* gene with polymorphic sites was subjected to PCR/restriction fragment length polymorphism (PCR-RFLP) analysis. Table 3 shows the frequencies of individuals with various genotypes and alleles at the *SAA1* locus in either FMF patients (n = 83) or Japanese healthy subjects (n = 200). The allele frequency of *SAA1.1* was significantly lower in FMF patients compared with healthy subjects (21.7% versus 34.0%). Conversely, the allele frequency of *SAA1.3* was higher in FMF patients compared with healthy subjects (48.8% versus 37.5%).

**Table 3:** Frequencies of the genotypes and alleles at the *SAA1* locus of Japanese patients with FMF and healthy subjects.

	FMF patients	Healthy subjects	*p* value
	n = 83(%)	n = 200(%)	
Genotype at *SAA1* locus			
1.1/1.1	4(4.8)	24(12.0)	χ2 = 12.553 *p* = 0.028
1.1/1.3	22(25.6)	49(24.5)	
1.1/1.5	6(7.2)	39(19.5)	
1.3/1.3	15(18.1)	27(13.5)	
1.3/1.5	29(34.9)	47(23.5)	
1.5/1.5	7(8.4)	14(7.0)	
Allele at *SAA1* locus			
1.1	36(21.7)	136(34.0)	χ2 = 9.563 *p* = 0.008
1.3	81(48.8)	150(37.5)	
1.5	49(29.5)	114(28.5)	

*SAA1*; Serum amyloid A1. Chi-square test was used to examine differences of genotype and allele frequencies between FMF patients and healthy subjects.

The -13C/T polymorphism, in the 5′-flanking region of the *SAA1* gene is associated with the *SAA1.3* allele and susceptibility to amyloidosis in Japanese RA patients [17]. We analyzed the frequency of -13C/T polymorphisms in FMF patients and Japanese healthy subjects. Allele frequencies of -13C/T were different among these two groups (Table 4), and -13T allele was significantly increased in FMF patients compared with healthy subjects (56.0% versus 41.0%, *p* = 0.001). These data suggest that the -13T allele is associated with susceptibility to FMF in the Japanese population. Allele frequencies of -13 C/T polymorphisms were also analyzed in typical or incomplete FMF patients. There was no significant difference in the frequencies -13T allele between typical and incomplete FMF patients (Table 5). Among 83 patients with FMF, 30 patients had 0 to 1 *MEFV* mutation (no mutation 3; heterozygous 27) and 53 patients at least 2 mutations (homozygous or compound heterozygous). There was no significant difference in *SAA1* gene polymorphisms between FMF patients with different numbers of *MEFV* mutations (Table 6).

**Table 4:** Frequencies of the genotypes and alleles at -13C/T *SAA1* locus of Japanese patients with FMF and healthy subjects.

	FMF patients	Healthy subjects	*p* value
	n = 83(%)	n = 200(%)	
Genotypes at -13C/T *SAA1*			
C/C	13(15.7)	67(33.5)	χ^2^ = 11.538 *p* = 0.003
C/T	47(56.6)	102(51.0)	
T/T	23(27.7)	31(15.5)	
Alleles at -13C/T *SAA1*			
T	93(56.0)	164(41.0)	χ^2^ = 10.682 *p* = 0.001
C	73(44.0)	236(59.0)	

*SAA1*; Serum amyloid A1. Chi-square test was used to examine differences of genotype and allele frequencies between FMF patients and healthy subjects.

**Table 5:** Allele frequencies of *SAA1* gene polymorphisms in typical and incomplete FMF patients.

	FMF criteria		*p* value
	Typical	Incomplete	
	2n = 92(%)	2n = 74(%)	
Allele at *SAA1* locus			
1.1	16(17.4)	20(27.0)	χ^2^ = 3.733 *p* = 0.155
1.3	44(47.8)	37(50.0)	
1.5	32(34.8)	17(23.0)	
Alleles at -13C/T *SAA1*			
T	51(55.4)	42(56.8)	χ^2^ = 0.029 *p* = 0.865
C	41(44.6)	32(43.2)	

**Table 6:** Number of *MEFV* gene mutations and *SAA1* gene polymorphisms in FMF patients**.**

	Number of mutations		*p* value
	0∼1 mutations	≧2 mutations	
	2n = 60(%)	2n = 106(%)	
Allele at *SAA1* locus			
1.1	11(18.3)	25(23.6)	χ^2^ = 0.955 *p* = 0.620
1.3	29(48.3)	52(49.1)	
1.5	20(33.3)	29(27.4)	
Alleles at -13C/T *SAA1*			
T	33(55.0)	60(56.6)	χ^2^ = 0.040 *p* = 0.841
C	27(45.0)	46(43.4)	

### Hardy-Weinberg equilibrium test

Finally, Hardy-Weinberg equilibrium was estimated by chi-square test with Yates' correction. There was no significant difference between observed and experienced frequencies of each genotype (*SAA1* -13C/T, *SAA2, IL-1β-511*) in the both FMF patients (Table 7) and healthy subjects (Table 7). These results indicated that these populations had a relatively stable genetic

**Table 7:** Frequencies of *SAA1* -13C/T, *SAA2*, *IL1β* -511 genotypes in Japanese patients with FMF and frequencies of *SAA1* -13C/T, *SAA2*, *IL1β* -511 genotypes in healthy subjects.

Frequencies of *SAA1* -13C/T, *SAA2*, *IL1β* -511 genotypes in Japanese patients with FMF				
Locus	Genotype	Observed number(%)	Expected number^a^	*p* value
*SAA1* -13C/T	C/C	13(15.7)	16.1	χ^2^ = 1.292 *p* = 0.256
	C/T	47(56.6)	40.9	
	T/T	23(27.7)	26.1	
*SAA2*	A/A	62(74.7)	61.6	χ^2^ = 0.007 *p* = 0.932
	A/G	19(22.9)	19.8	
	G/G	2(2.4)	1.6	
*IL1β* -511	C/C	27(32.5)	28.3	χ^2^ = 0.144 *p* = 0.704
	C/T	43(51.8)	40.3	
	T/T	13(15.7)	14.3	

Frequencies of *SAA1* -13C/T, *SAA2*, *IL1β* -511 genotypes in healthy subjects				
Locus	Genotype	Observed number(%)	Expected number^a^	*p* value
*SAA1* -13C/T	C/C	67(33.5)	69.6	χ^2^ = 0.384 *p* = 0.535
	C/T	102(51.0)	96.8	
	T/T	31(15.5)	33.6	
*SAA2*	A/A	163(81.5)	162.9	χ^2^ = 0.104 *p* = 0.747
	A/G	35(17.5)	35.2	
	G/G	2(1.0)	1.9	
*IL1β* -511	C/C	59(29.5)	59.4	χ^2^ = 0.001 *p* = 0.978
	C/T	100(50.0)	99.2	
	T/T	41(20.5)	41.4	

a Expected genotype frequencies based on observed allele frequencies and assuming Hardy-Weinberg equilibrium.

background and were stable for genetic statistical analysis.

## Discussion

FMF is considered to be an autosomal recessive disease [18]. The gene causing FMF is *MEFV*, which encodes pyrin, expressed in the cytoplasm of myeloid cells [2]. Pyrin is postulated to act as a negative regulator of IL-1-mediated inflammation [19]. However, approximately 30% of FMF patients exhibit a single *MEFV* mutation, despite sequencing of the entire *MEFV* genomic region and other autoinflammatory genes [20]. More recently it was demonstrated that pyrin truncation in mice did not show an overt phenotype of FMF, however, pyrin-deficient and FMF-associated B30.2 mutations “knock in” mice showed severe spontaneous inflammatory phenotype, suggesting that FMF may be caused by a gain of function by disease-associated missense changes in pyrin and that FMF may not be a pure autosomal recessive disease due to the loss of protein function [21]. One explanation is that subjects having a single *MEFV* mutation may develop an FMF phenotype in the presence of other inflammasome-related genes or in the presence of other environmental factors [22]. Therefore, the role of potential modifier genes and polymorphisms within these gene families should be assessed in conjunction with genotype-phenotype association studies. Polymorphisms in genes associated with the inflammasome pathway can affect the development of FMF [5]. For example, TLR2 polymorphisms may be an important factor in the susceptibility of FMF [23], [24].

In this study, we investigated the *SAA1* and *IL-1β* gene polymorphisms in Japanese patients with FMF. There was no significant difference in *IL-1β-511* (C/T) or *IL-1Ra* VNTR polymorphisms between FMF patients and healthy subjects in accord to the previous report [25]. However, we demonstrated that *SAA1* gene polymorphisms, which are attributed to AA amyloidosis, might be also responsible for susceptibility to FMF. It is clear that genotypes at the *SAA1* locus are associated with an increased susceptibility to AA amyloidosis [26]. However, the contribution of these genotypes to the occurrence of non-amyloid, inflammatory disease has not been elucidated. In this study, we investigated the allele frequencies of *SAA1.1* and -13 (C/T) polymorphisms of the *SAA1* promoter region in Japanese patients with FMF. Our data demonstrated that the -13T allele polymorphism was a major risk factor and that the *SAA1.1* allele was protective for the occurrence of FMF in Japanese case-control studies.

The presence of 2 single-nucleotide polymorphisms (SNPs) within exon 3 of the *SAA1* gene, 2995 C/T and 3010 C/T, defined 3 haplotypes that corresponded to the *SAA1.1*, *SAA1.3*, and *SAA1.5* isoforms [26]. In Japanese patients with RA, homozygote expression of the *SAA1.3* allele was a proven risk factor, whereas *SAA1.1* appeared to be protective for AA amyloidosis [27]. In contrast, a strong positive association with *SAA1.1* has been established in Caucasian patients with amyloidosis secondary to juvenile idiopathic arthritis and FMF [28]–[30]. Moriguch *et* *al.* identified another *SAA1* SNP, the -13T/C SNP in the 5′-flanking region of the *SAA1* gene [17]. They observed the -13T allele was associated with AA amyloidosis, and associated with the *SAA1.3* allele in Japanese RA patients [17]. Interestingly, a polymorphism in the *SAA1* promoter -13T allele was found to be significantly associated with increased AA amyloidosis risk in both populations and to be in linkage disequilibrium with *SAA1.1* and *SAA1.3* in Caucasian and Japanese patients, thus apparently explaining the previous discrepancy [31]–[35]. Functional studies have demonstrated that the -13T allele is responsible for a higher transcriptional rate [36]. However, this did not result in higher serum levels of SAA, possibly due to increased proteolytic processing rates of *SAA1.1* and *SAA1.3* compared to *SAA1.5* [37]. The mechanisms by which the -13T allele predisposes to FMF remains to be unraveled and many possibilities have been suggested.

The overproduction of IL-1β, induced by NLRP3 inflammasome activation, is responsible for a variety of autoinflammatory syndrome including FMF. The NLRP3 inflammasome has emerged as a critical cytosolic sensor for a number of endogenous mediators, including amyloid proteins [6]. Recent studies indicated that SAA activates the NLRP3 inflammasome in a cathepsin B and P2X_7_-dependent manner, resulting in the secretion of mature IL-1β [10]. The accumulation of newly formed AA amyloid fibrils and aberrant processing of SAA is relevant to AA amyloidogenesis [38]. Therefore, in subjects with AA amyloidogenic genetic factors, such as -13T allele, the presence of SAA-derived AA amyloid fibrils may implicate the NLRP3 inflammasome activation pathway, which is thought to be relevant to the pathogenesis of FMF. Jeru *et al*. demonstrated that the *SAA1* genotype influenced the severity of FMF and disease susceptibility through a negative selection process, providing new insights into the role of *SAA1* in the pathophysiology of FMF [39]. Assuming that *SAA1* gene polymorphisms induce the formation of AA amyloid fibrils, this suggests that the polymorphisms may be associated with the NLRP3 inflammasome activation process and susceptibility to FMF. These findings may provide insights into modifier factors, other than *MEFV*, in the development of FMF.

The gender discrepancy (female dominant in incomplete FMF) seen in the present study may result from hormonal or associated environmental factors, which generate a disease of atypical or milder severity in female. For example, the risk for developing amyloidosis had been shown to be higher in male patients with FMF [40], [41]. These findings suggest that clinical variability observed in FMF may be party attributed to the influence of environmental factors including gender. The main limitations of the study are its localization to a certain country, and a limited number of patients.

In occlusion, this study shows a significant prevalence of the -13T allele in Japanese patients with FMF. This comparative case-control study demonstrated that the *SAA1* gene polymorphisms might affect susceptibility to FMF, which is presumed to be a monogenic disease. Further studies are required to determine the impact of *SAA1* gene polymorphisms and the occurrence of FMF in large studies in different geographic areas.

### Ethics approval

This study was conducted with the approval of the ethical committees of Nagasaki Medical Center.
